# Regions of Homozygosity in the Porcine Genome: Consequence of Demography and the Recombination Landscape

**DOI:** 10.1371/journal.pgen.1003100

**Published:** 2012-11-29

**Authors:** Mirte Bosse, Hendrik-Jan Megens, Ole Madsen, Yogesh Paudel, Laurent A. F. Frantz, Lawrence B. Schook, Richard P. M. A. Crooijmans, Martien A. M. Groenen

**Affiliations:** 1 Animal Breeding and Genomics Group, Wageningen University, Wageningen, The Netherlands; 2 Department of Animal Sciences, University of Illinois at Urbana-Champaign, Urbana, Illinois, United States of America; The University of Queensland, Australia

## Abstract

Inbreeding has long been recognized as a primary cause of fitness reduction in both wild and domesticated populations. Consanguineous matings cause inheritance of haplotypes that are identical by descent (IBD) and result in homozygous stretches along the genome of the offspring. Size and position of regions of homozygosity (ROHs) are expected to correlate with genomic features such as GC content and recombination rate, but also direction of selection. Thus, ROHs should be non-randomly distributed across the genome. Therefore, demographic history may not fully predict the effects of inbreeding. The porcine genome has a relatively heterogeneous distribution of recombination rate, making *Sus scrofa* an excellent model to study the influence of both recombination landscape and demography on genomic variation. This study utilizes next-generation sequencing data for the analysis of genomic ROH patterns, using a comparative sliding window approach. We present an in-depth study of genomic variation based on three different parameters: nucleotide diversity outside ROHs, the number of ROHs in the genome, and the average ROH size. We identified an abundance of ROHs in all genomes of multiple pigs from commercial breeds and wild populations from Eurasia. Size and number of ROHs are in agreement with known demography of the populations, with population bottlenecks highly increasing ROH occurrence. Nucleotide diversity outside ROHs is high in populations derived from a large ancient population, regardless of current population size. In addition, we show an unequal genomic ROH distribution, with strong correlations of ROH size and abundance with recombination rate and GC content. Global gene content does not correlate with ROH frequency, but some ROH hotspots do contain positive selected genes in commercial lines and wild populations. This study highlights the importance of the influence of demography and recombination on homozygosity in the genome to understand the effects of inbreeding.

## Author Summary

Small populations have an increased risk of inbreeding depression due to a higher expression of deleterious alleles. This can have major consequences for the viability of these populations. In domesticated species like the pig that are artificially selected in breeding populations, but also in wild populations that experience habitat decline, maintaining genetic diversity is essential. Recent advances in sequence technology enabled us to identify patterns of nucleotide variation in individual genomes. We screened the full genome of wild boars and commercial pigs from Eurasia for regions of homozygosity. We found these regions of homozygosity were caused by the demographic history and effective population size of the pigs. European wild boars are least variable, but also European breeds contain large homozygous stretches in their genome. Moreover, the likelihood of a region becoming depleted depends on its position in the genome, because variation has a high correlation with recombination rate. The telomeric regions are much more variable, and the central region of chromosomes has a higher chance of containing long regions of homozygosity. These findings increase knowledge on the fine-scaled architecture of genomic variation, and they are particularly important for population genetic management.

## Introduction

The effect of parental relatedness on the fitness of the offspring has long been recognized. Consanguineous matings cause the inheritance of haplotypes that are Identical By Descent (IBD), resulting in potentially long homozygous stretches across the genome of the offspring. These Regions Of Homozygosity (ROHs) increase the risk of recessive deleterious alleles to be co-expressed, reducing the viability of the organism. In human and canine populations, large homogeneous outbred populations have a lower proportion of genomic autozygosity than small isolated populations [1], [2], [3]. In addition, studies have shown a correlation between homozygous stretches in the genome and human diseases [4], [5], [6]. One of the long standing interests across various facets of biology is to understand the direct consequences of inbreeding. The inbreeding coefficient F is a commonly used statistic to estimate the degree of same alleles inherited as a consequence of parental relatedness [7]. However, inbreeding depression may greatly vary across the genome and studies using few molecular markers are unlikely to detect these differences. Thus, it is important to understand the genomic distribution of IBD alleles, to fully grasp the importance of inbreeding for the viability of a given population. The biological characteristics of a species, such as mating systems and reproductive rate, play an important role in maintaining genetic diversity in a population. Moreover, the interactions between standing genetic variation, and past and current demography effect the degree of inbreeding in a population. Homozygosity is used in artificial mate selection to minimize progeny inbreeding [8]. Maintenance of the minimum viable population size (MVP) is essential for a population to ensure its persistence in time [9]. This is important for conservation efforts but also in commercial breeding. But, the intrinsic features of the genome that contribute to its architecture, such as recombination rate, are usually neglected in estimations of genetic variation and associated considerations for genetic conservation [10], [11].

In a randomly mating population, IBD tracts are expected to be broken down through time by recombination. In humans, ROH decay is thought to follow an inverse exponential distribution with each generation since the common ancestor halving the ROH size [12], [13]. Thus, the size and position of ROHs in the genome are expected to correlate with recombination rate [14]. Homozygous stretches should be non-randomly distributed if, as is expected, recombination rate varies throughout the genome and cannot be explained only by past demography. The occurrence of ROHs should rather be an interaction between demography and the recombination landscape. [15] showed that ROHs may have swept through a population because of positive selection of a particular allele in the region. Moreover, ROHs derived from consanguineous mating may falsely appear to be a signature of positive selection, as these two effects are expected to display depletion of polymorphism in a given genomic region. Therefore, it is important to understand how ROHs segregate across the genome if we are to distinguish signal of selection from inbreeding.

Previous studies that investigated the pattern of ROHs in different mammalian species found that the occurrence of ROH correlates with recombination rate [16], [17]. However, These studies were based on homozygosity scores from high-density single nucleotide polymorphism (SNP) chips. Recent advances in sequencing technology enable a thorough investigation of genome-wide SNP distributions, and can largely extend the use of high-density SNP arrays for ROH identification. Moreover, re-sequencing strategies should enable a less biased characterization of variation, whereas SNP chips usually suffer from ascertainment bias. In addition, subtle effects of recombination rate can be examined with a full genomic resolution. The porcine genome is known to have a relatively heterogeneous distribution of recombination rate and GC content [18]. Particularly the central parts of chromosomes have a much lower recombination rate than peripheral parts. Although this effect is present in other mammalian genomes, it seems much more pronounced in the porcine genome. In In addition, the species *S. scrofa* (domestic pigs and Eurasian wild boars) is known to have very diverse population structure across its natural and artifical habitat. These characteristics make *Sus scrofa* an excellent model to study the effect of recombination and demography on the distribution of ROHs in mammalian genomes.

The genus *Sus* originated in Southeast Asia during a speciation event in the late Miocene or near the Miocene/Pliocene boundary ∼14-4 million years ago (Mya) ([19], [20], [21], Frantz LAF et al., unpublished data), and the wild boar expanded its range all throughout Eurasia in the Pleistocene ∼1 to 0.5 Mya [22]. The European wild boar populations, which are geographically the most distal from the putative origin of the species, are thought to have separated from Southeast Asian *Sus scrofa* in the late Pleistocene between 0.5 and 0.9 Mya [19], [22], [23]. The latest glaciation events in Europe created population bottlenecks and subsequent post glacial demographic expansion from refugia in the Iberian Peninsula and the Balkans [23]. The genetic diversity of Asian wild boars was probably less affected by the latest glaciation event because a larger area of suitable habitat would have remained available, although it may have separated Northeastern and Southeastern wild boars [24]. The pig has been domesticated at least twice, independently, from local wild boar populations in Asia Minor and China around 8,000 years ago, and there was probably recurrent introgression from the wild species and between breeds since the first domestication event [25]. Because of possible introgression, or even *de novo* domestication, Near Eastern mitochondrial haplotypes have been completely replaced by European wild boar haplotypes in European commercial pigs [19]. Known population histories of *Sus scrofa*, both wild and domesticated, provide a valuable framework for population genomic studies, as conclusions from sequence data can be supported by demography.

This study uses re-sequencing data for the analysis of ROHs and nucleotide diversity (π, [26]), to explore how genomic distribution of ROH and π is shaped by additive effects of the recombination landscape, demography and selection. The polymorphism distribution in complete genomes from multiple individual pigs, from different breeds and wild populations from Europe and Asia, are studied in substantial detail. We expect the abundance of ROHs in the genome to be correlated to effective (past and current) population size. The size of ROHs in particular can be expected to correlate to recent and current population size, reflecting founder effects and population bottlenecks. Nucleotide diversity between non-IBD haplotypes, should rather reflect past or ancient population size. In addition, we investigate the influence of recombination rate on the genomic ROH patterns. This highly heterogeneous genomic recombination landscape make pigs and wild boar very well suited for studying the effects of recombination on shaping variation on a genome-wide scale. Furthermore, we investigate the integral effects of demography and recombination on the distribution of ROHs. Finally, we investigate ROH hotspots for traces of positive selection and gene content. Since these different factors are interconnected, the formation and degradation of ROHs is a dynamic genomic process. Overall, we found that a combination of past demographic events and the recombination landscape mostly shaped the pattern of ROHs in the genome.

## Results

### General statistics

**Figure 1:**
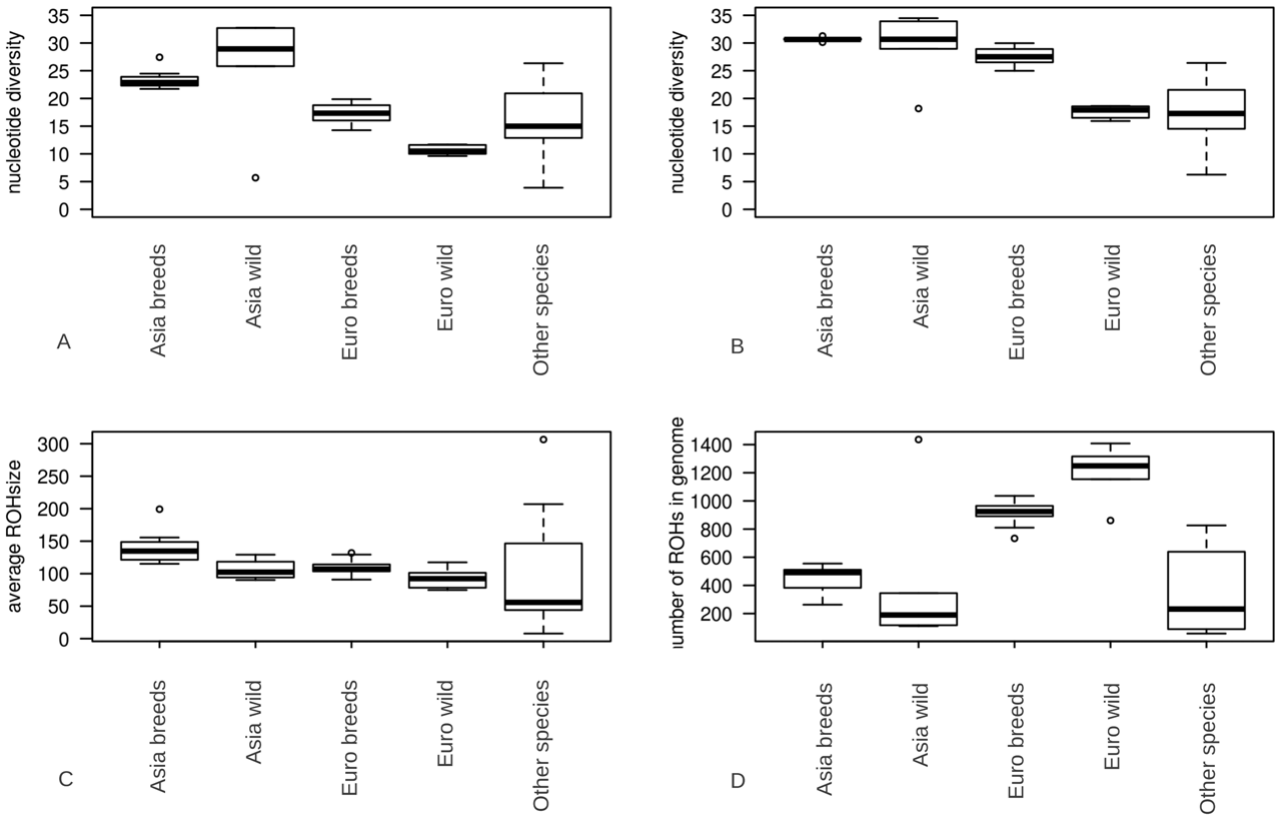
Summary statistics for genomic variation. The distributions of ROH statistics for Asian breeds (n = 7), Asian wild boars(n = 5), European breeds (n = 29), European wild boars (n = 6) and other species (n = 5). Groups are divided based on geography (Asians and Europeans), domestication (pigs and wild boars) and speciation (Other-species include the African Warthog *Phacochoerus africanus* and other representatives of the Sus genus being *Su*s *barbatus, Sus celebensis, Sus verrucosus and Sus cebifrons*). Values are averaged within individuals resulting in a single data point per ROH characteristic for each individual. 1A. nucleotide diversity including ROHs (*10^−4^ bp) 1B. nucleotide diversity excluding ROHs (*10^−4^ bp) 1C. Average ROH size (*10^4^ bp) 1D. number of ROHs in the genomes of individuals.

Regions of homozygosity in the autosomes of individuals were determined by re-sequencing pigs and wild boars of Asian and European origin. We grouped our samples based on geographic origin and domestication status for further analysis (Figure S1; Table S1). Pigs were separated into five groups, being Asian domesticated, Asian wild, European domesticated, European wild and other species. Grouping was based on geography and domestication rather than phylogeny. ROHs were identified in all 52 sequenced individuals (examples in Figure S2, details in Table S1). We found an average number of 778.8 ROHs/genome (+/−349) with an average size of 1.11 Mbp. ROH size ranged from 10 Kbp (minimum size considered) to 83.6 Mbp (29% of the chromosome). Genome-wide nucleotide diversity (π) was on average 1.733 SNPs/Kbp (+/−0.57) and 2.49 SNPs/bp (+/−0.57) in the genomic regions outside ROHs (π-out). The difference in π and π-out varied between 1.2 SNPs/Kbp in an European Large White pig, and 0.05 SNPs/Kbp in the *Sus barbatus* individual. The mean number and size of ROHs varied significantly between European and Asian domesticated pigs (p<0.001) as well as between wild boars and breeds within continents (p<0.001, Figure 1C and 1D). On average 23% of the genome was considered to be a region of homozygosity. Nucleotide diversity outside ROHs was not significantly different between domesticated pigs and wild boars within Asia, but did vary between the two continents and within the two European groups (p<0.001, Figure 1B). The most extreme ROH coverage was observed in the Japanese wild boar (78% of its genome).

### Effects of demography on ROH distribution

**Figure 2:**
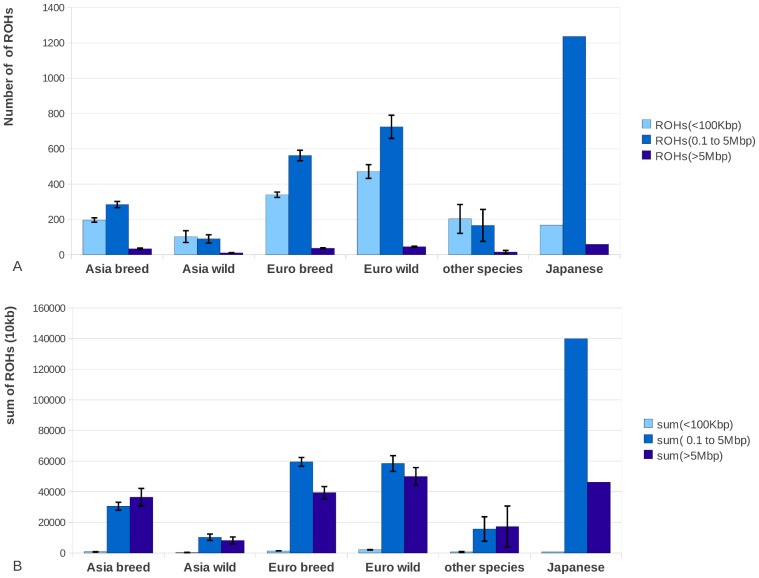
Total number of ROHs and proportion of the genome covered by ROHs. 2A. The average number of ROHs belonging to three size classes small (<100 Kbp) medium (0.1 to 5 Mbp) and large (>5 Mbp) for each of the different groups. 2B. The total size of the genome that is covered by the particular ROH size class in one individual, averaged per group. The Japanese wild boar is shown separately and is not included in the Asian group, as its demographic history from an island population and the associated ROH pattern is very distinct from all other sampled individuals. Asian wild boars (n = 4), Asian pigs (n = 7), European wild boars (n = 6), European pigs (n = 29), other species (n = 5).

ROHs were separated into three size classes: 1) small (<100 Kbp), 2) medium (0.1 to 5 Mbp) and 3) large (>5 Mbp). We computed the proportion of ROHs falling in each class in all our 52 samples. While small ROHs were abundant throughout the genome, their absolute contribution to the genome was relatively small (Figure 2). In contrast, medium sized ROHs were the less common but covered significantly more of the genome than small and large ROHs. The large ROHs were at least a tenfold less abundant than medium ROHs, but nevertheless covered a significant proportion of the genome. Asian domesticated pig genomes were covered mostly by large ROHs. Asian wild individuals had much fewer genomic ROHs and also a smaller proportion of ROHs in their genome than all European pigs and the Asian domesticated pigs (p<0.0001). European wild boars had on average the highest number of ROHs and highest proportion of genomic autozygosity. The Japanese wild boar is an outlier in both number of ROHs and cumulative size likely due to its island bio-geographical background, so we treated it separately. The divergence between the wild boars in Europe and Asia was estimated to have occurred ∼1.2 mya and a major drop in population size in both continental groups took place from ∼50 kya and onwards, based on individual genome demographic inference as implemented in the Pairwise Sequentially Markovian Coalescent (PSMC) model (Figure S3). Population size in the Asian *Sus scrofa* is thought to not have been reduced as severely as for the European populations, which is supported by the nucleotide diversity outside ROHs and ROH analysis (Figure 1A–1B and Figure 2). In addition, the Asian wild populations were estimated to have a larger effective population size than the Asian domesticated pigs, and the European wild populations had the smallest population size based on the ROH analysis.

**Figure 3:**
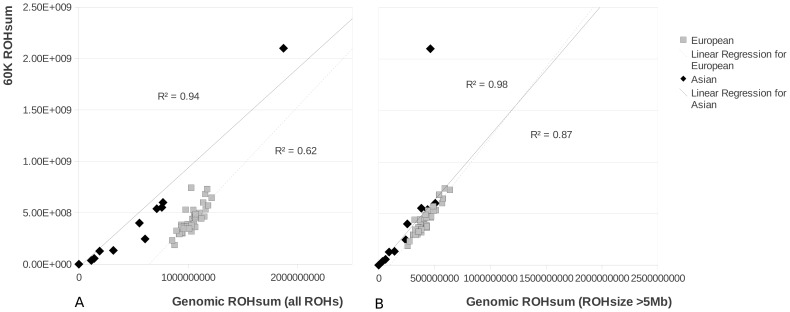
ROH size in pigs based on the 60K chip method and the re-sequencing method. 3A shows the correlation between both methods when the total sum of ROHs is taken from the re-sequencing method (‘Genomic ROHsum’) and the 60K chip method (‘60K ROHsum’). In 3B the correlation is shown when only the ROHs over 5 Mbp are taken into account for the re-sequencing method. The outlier for the Asian group (the Japanese wild boar) is not included in the R^2^ calculations.

We tested the utility of the Illumina porcine 60K beadchip to identify ROHs in the three size classes. Genotyping arrays are widely used and offer the possibility to cost-effectively study a much wider sample size and to test the usefulness of this technology for the detection of ROHs. Using this array we evaluated whether the results from whole genome re-sequencing analyses for a limited number of individuals could be extrapolated to an entire population. The chip-based methodology was capable of detecting the ROHs larger than 5 Mbp (Figure 3) but underestimated the cumulative size of ROHs in the genome, especially for the European samples. This phenomenon is likely to be due to the number of small sized ROHs in European populations, which cannot be detected due to the limited resolution of the SNP chip. The Japanese wild boar had many ROHs, but the ROH size was not extremely large because the ROHs were interceded by short sections with variable sites (Figure S2 and Table S1). Therefore, the total sum of ROHs was probably overestimated in this individual (Figure 3) by the chip-based method and showed a weak correlation with the cumulative ROH size of >5 Mbp homozygous blocks that were identified with the re-sequencing method. Since ROHs in the highest size class are fully detected (>5 Mbp, Figure 3) comparing populations based on their 60K-defined ROH distribution is valid for analyis of large ROHs. Naturally, the limited capability of detecting shorter ROHs has implications for the inferred demography and therefore we use the 60K defined ROHs only for comparison with our largest sequence based ROHs.

**Figure 4:**
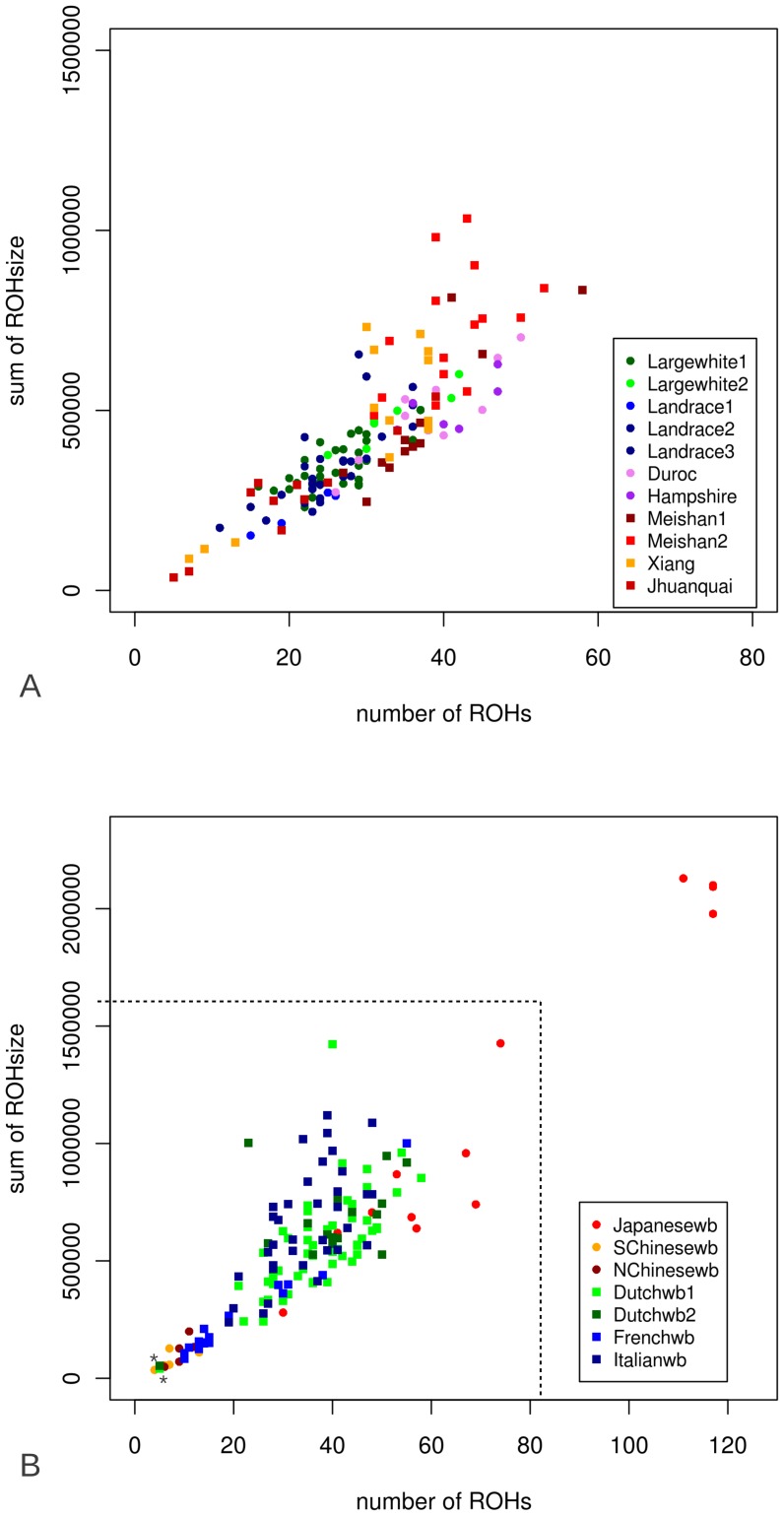
Number and cumulative ROH size (ROHs>5MB) for all genotyped individuals. Number of ROHs and sum of ROHs detected by PLINK for all 241 individuals genotyped by the Illumina porcine 60k beadchip. Sum of ROHsize is *1000 bp. 4A. ROHs in domesticated individuals. Asian pigs are shown in red, orange and purple and the European pigs are in blue and green. 4B. ROHs in wild individuals. Asian wild boars are shown in red and orange and the European wild populations are displayed in green and blue. The dashed line represents the range of ROH number and ROH size for the domesticated individuals. The individuals marked with * are putative hybrids.

241 individuals from the different *Sus scrofa* populations that had been re-sequenced were genotyped using the 60K assay, and number and cumulative ROH size where scored. Details of the genotyped individuals can be found in Table S2. Sequenced individuals were never extreme within their population in terms of ROH number or ROH size. In the Asian and European breeds, the number of ROHs ranged from 5 to 59 and cumulative ROH size was 10 Mb to 1 Gb (Figure 4). European breeds had a narrower distribution of number of ROHs and cumulative ROH size. Both sum and number of ROHs in the Asian breeds Jianquhai and Xiang showed a modest bimodal distribution. The Chinese wild boars tended to have fewer ROHs and cumulative ROH size than their European relatives. Even though cumulative ROH size for the Japanese wild boars may have been overestimated because of the low resolution of the 60K chip, four individuals were extremely homozygous with more than 2/3 of their genome consisting of ROHs. Variances in ROH size and abundance in the Japanese wild individuals was much higher than in the other groups. Notable, two Dutch wild boars had significantly fewer ROHs than all other European wild boars from the same populations (indicated with an* in Figure 4B).

**Figure 5:**
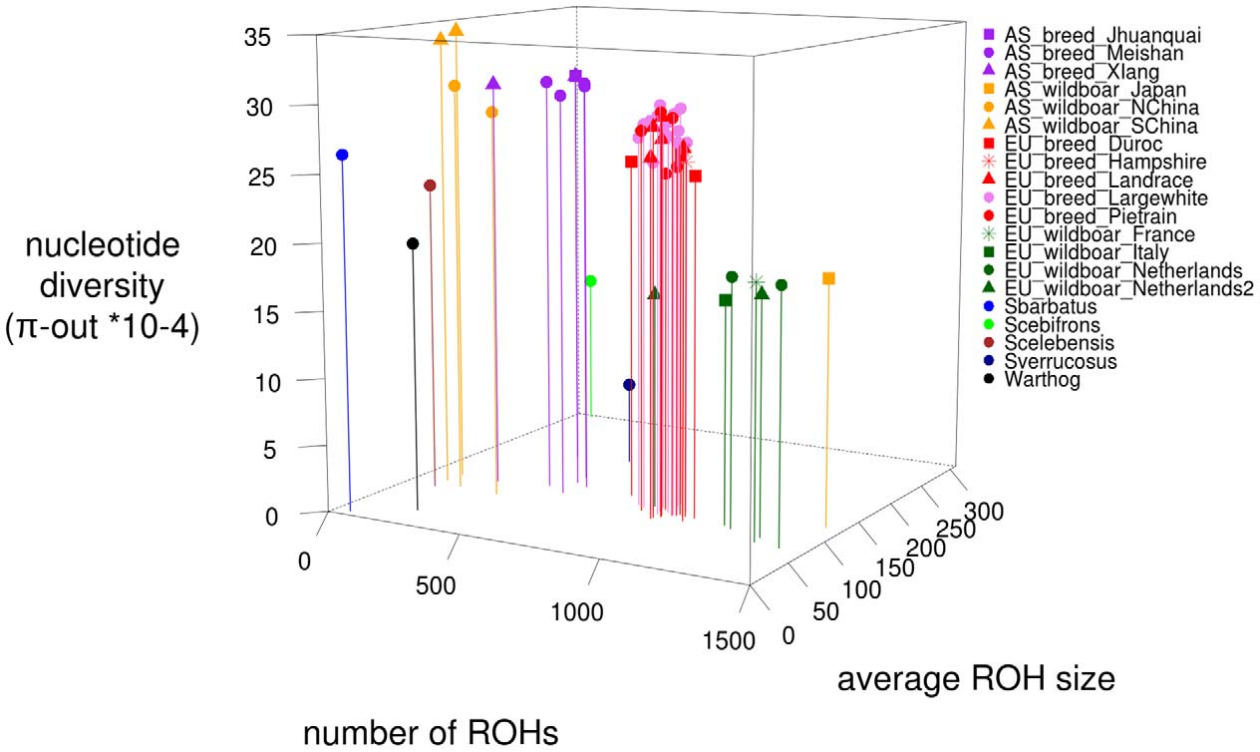
Three-point ROH statistics for all 52 sequenced individuals. On the x-axis, the number of ROHs in the genome per individual is plotted, the average ROH size (*10^4^ bp) is displayed on the y-axis and the nucleotide diversity outside ROHs in a 10 kb window ‘nucleotide diversity (π-out *10^−4^)’ on the z-axis. Coloration is based on relatedness and geography, with individuals from the same populations having the same color.

The genomic variation pattern for the 52 re-sequenced individuals was analyzed in more depth. We found that statistics such as π-out (nucleotide diversity outside ROHs), average ROH size and total number of ROHs were good predictors to assign individual to their corresponding group (Figure 5). Interestingly, while all Asian wild boars formed a monophyletic clade on our phylogenetic tree (Figure S1), we found that the Japanese sample did not cluster with other Asian samples on our three dimensional plot (Figure 5). The Chinese wild boars represent the most variable cluster due to their high nucleotide diversity and few ROHs (p<0.001). We found that nucleotide diversity was higher in European breeds than European wild boars (p<0.0001). Moreover, total number of ROHs in the genome was also lower in European breeds. This resulted in two clusters in our three dimensional plot (Figure 5), contrasting with the monophyletic status of European populations on our phylogenetic tree (Figure S1). The Asian breeds were more inbred than their wild ancestors but displayed fewer ROHs and higher nucleotide diversity than the European animals (p<0.0001). The sequenced *Sus verrucosus* individual had the lowest genomic variation of all tested animals due to extremely low nucleotide diversity, intermediate ROH number and large ROH size. The sequenced *Sus barbatus* individual had the least ROHs and smallest ROH size of any of the sequenced individuals, suggesting the individual is highly outbred, with a high effective population size. The ROH pattern in *Sus cebifrons* was particularly interesting because the total number of ROH was very low, but it contained few very large ROHs and had relatively low nucleotide diversity.

### Effects of recombination rate on ROH distribution

**Figure 6:**
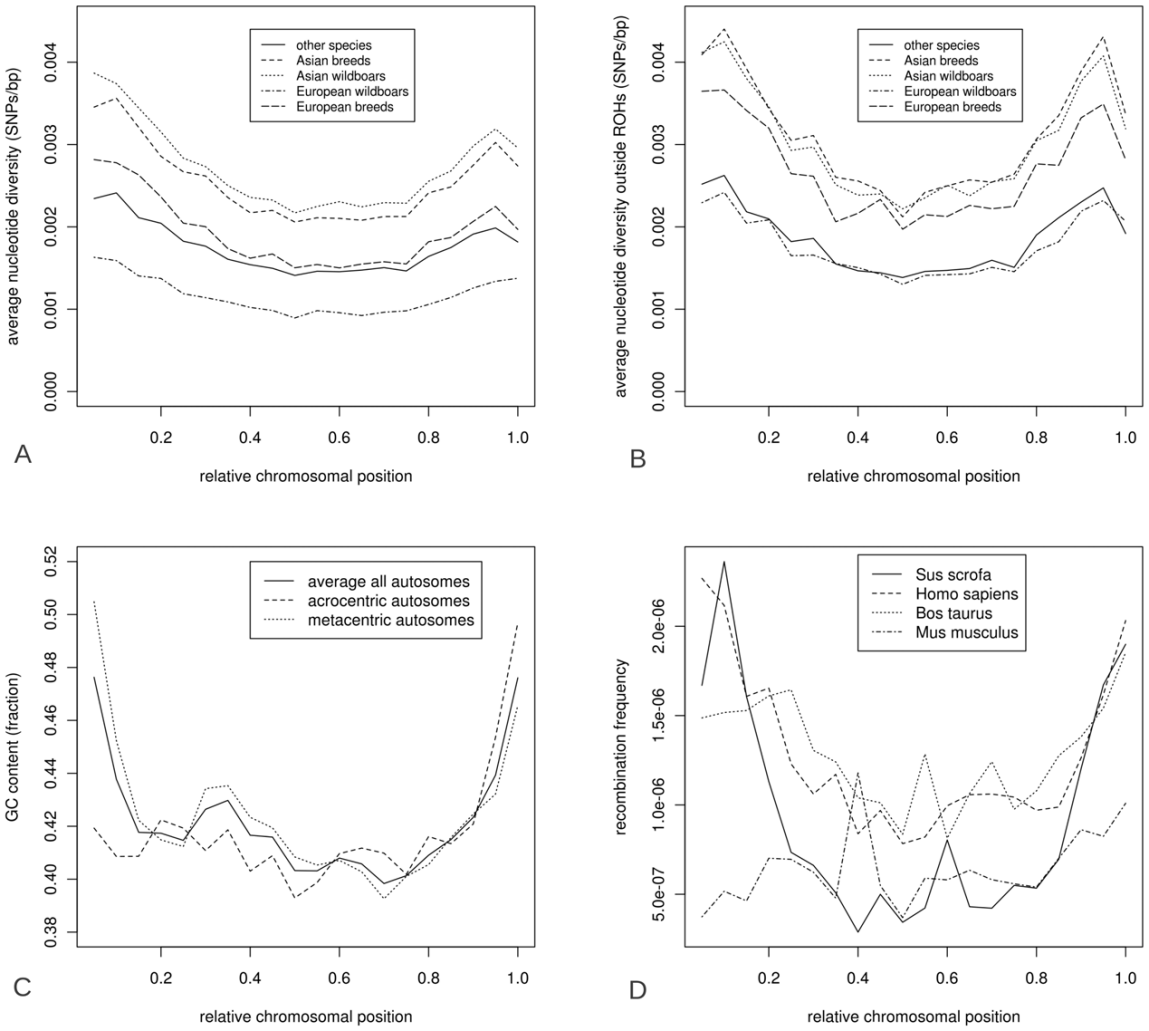
Characteristics of the porcine genome over relative chromosomal position. Physical distribution of total nucleotide diversity (6A), nucleotide diversity outside ROHs (6B), GC content (6C), recombination rate (6D) over chromosomes. Relative chromosomal position is averaged for all chromosomes so that 0.0 represents the left telomeric region and 1.0 the far right telomere.

To test for the effect of recombination rate and GC content on ROH formation and distribution, we computed GC content and recombination rate relative to the physical chromosomal position, for each chromosome separately, and averaged the results over all chromosomes (Figure 6C and 6D). The GC content was based on the porcine reference genome build 10.2 [21]. GC content was generally higher when moving toward telomeric regions in metacentric chromosomes and toward chromosomal edge in acrocentric chromosomes (Figure 6A). Overall, GC content was inversely correlated with distance to the telomeres (Figure 6C). Recombination rate for pigs was calculated based on ∼60.000 markers, obtained from Tortereau et al and averaged over all chromosomes [18]. Variation in recombination fraction over the physical position of the chromosomes, with high recombination rates at telomeric regions and very low recombination rates at the central part of chromosomes, was most pronounced in pigs and virtually absent in mice (Figure 6D). A ‘U-shaped’ distribution of recombination rates was present in all chromosomes in pigs, while in humans this is only observed in metacentric chromosomes (data not shown). Nucleotide diversity correlated strongly with both recombination rate (cor = 0.88, p<0.00001) and GC content (cor = 0.61, p<0.005). nucleotide diversity greatly increased in the European breeds and wild boars when ROH bins were excluded. However, this phenomenon was only observed in Asian breeds at the chromosome tips (Figure 6A, 6B). ROH distribution was negatively correlated with GC content, recombination rate and nucleotide diversity outside ROHs (cor = −0.71, −0.87 and −0.95 respectively, p<0.0001 for all). This is expected as these genomic features all appeared to be highly correlated.

**Figure 7:**
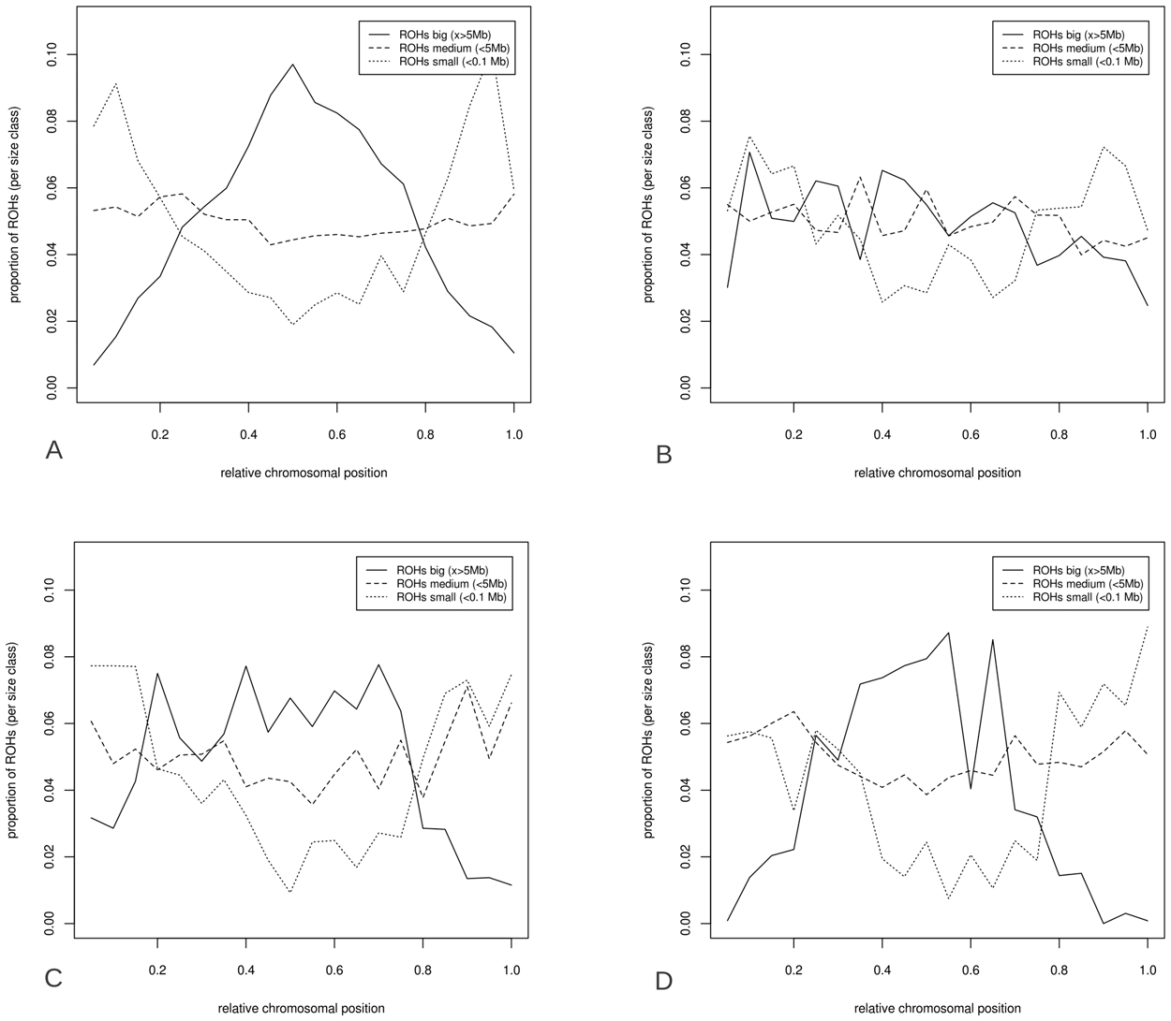
Distribution of ROHs over relative chromosomal position. ROHs were split into three size classes; big (x>5 Mbp), medium (0.1 to 5 Mbp) and small (x<0.1 Mbp). The distribution is relative only to the total number of ROH bins in that particular size class, and the distributions are averaged over all chromosomes. ROH distribution is given for four groups: European pigs (7A), European wild boars (7B), Asian pigs (7C) and Asian wild boars (including the Japanese, 7D).

The likelihood of ROH occurrence at a particular chromosomal position was dependent on the size of the ROH (Figure 7). The ROHs from the four *Sus scrofa* groups were separated into the three previously mentioned size classes (small, medium, big) and the relative distribution of ROHs over the genome was calculated for each size class (the Japanese wild boar is included in the Asian wild boar group, Figure 7D). The largest ROHs appeared more in the low recombination regions in the middle of the chromosome in European breeds and both Asian groups, and the smallest ROHs had a relatively higher distribution towards the telomeric regions (p<0.001). Medium sized ROHs seemed to be evenly distributed across the genome in all groups. The ROHs in European wild boars tend to be more evenly distributed than those in other groups (Figure 7B). The differences in ROH occurrence and nucleotide diversity between the extreme regions in recombination frequency were most profound in the European domesticated pigs.

### ROH, gene content, and shared homozygosity

We investigated the correlation of gene content and ROH occurrence on a genome-wide scale. We found no significant correlation between gene content and ROH count. This result was confirmed when repeating the same analysis in different sample groups (Figure S4). Moreover this was still not significant when using different classes of ROHs (small, medium, large). No regions of homozygosity overlapped in all sequenced *Sus scrofa* individuals. We found two overlapping ROHs among our European breeds (cumulative size of 6.03 Mbp). The first region on chromosome 1 did not contain any genes, whereas the second region on chromosome 9 contained 11 genes, of which 7 were semaphorins. These genes areinvolved in cell differentiation (nervous system development) and have previously been identified as differentially expressed between the Large White breed and Iberian pigs [27].

Among all homozygous regions in the pigs from Asian breeds, 4 regions were shared among all individuals with a cumulative size of 4,38 Mbp. The largest region was on chromosome 1 and contains a total of 1136 genes. Two neighboring fractions of chromosome 3 contained a ROH in all Asian breeds. Interestingly, we found that one of these regions also partially overlapped with ROHs found in all Asian wild boars, but not in all European pigs. This gene dense region contained 91 genes total, and has previously been reported as a region possibly under positive selection in Asian pigs [21]. The last homozygous region in the Asian breeds on chromosome 5 contained 3 genes including LEMD3, MSRB3, which is involved in oxidation reduction, and the WIF1 gene, involved in positive regulation of fat cell differentiation.

We found 28 ROHs that were overlapping among our 13 Large White samples (European breed; cumulative size of 54.38 Mbp). All of these ROHs were carrying genes (336 genes in total). GO analysis revealed 37 significantly overrepresented GO-IDs, o.a. involved in the organization and assembly of nucleosome and involved in reproduction (Table S3). ROHs that were found exclusively in all individuals in the Large White – Landrace group of breeds contained 11 genes, including the PLAG1 gene on chromosome 4, which is related to growth.

The European wild boars displayed shared genomic homozygosity on a total of 47,52 Mbp in 81 regions. Out of these mutually homozygous regions, only 26 were carrying genes. The 97 genes in these regions contributed to 24 significantly overrepresented GO-IDs. Some enriched terms found in the Large Whites were also overrepresented in the European wild boars, mostly histones. Histones are usually very sytenic, which may explain the clustering of histone related genes and may not have any functional relevance. Macromolecular complex and cellular component assembly and organization were abundant in the list for wild boars, as well as defense responses (Table S4).

## Discussion

Recombination maps are currently available for only a few vertebrate species. The pig shows very pronounced differences in recombination-rate throughout the genome, and highly diverse demographies in both wild and domesticated populations, making it an excellent model species to demonstrate the interaction between recombination landscape and demography.

### Effects of demography

The size and abundance of ROHs in the porcine genome varied markedly between individuals from different (sub) populations. Animals from the same population tend to have similar ROH patterns in their genome (Figure 4 and Figure 6), which indicates of the influence of shared demography on ROH distribution. The class of large ROHs is most sensitive to recent population changes. Thus, the bimodal distribution of large ROHs in Asian breeds may be explained by the sampling of two different populations. In European humans, the number and expanse of ROHs correlated strongly with latitude [13] but not longitude. Kirin et al. also showed a correlation between geography and genomic ROH occurrence in humans [28]. In our study we also showed a significant difference in ROH abundance and size between European and Asian wild boars. However, the Japanese wild boar shared a long demographic history with the other Asian wild boars compared to all other individuals, and yet displayed a completely different ROH pattern. The ROH pattern in this individual is consistent with a small current population size. The high number of ROHs (rather than ROH size or nucleotide diversity) indicates that the population has been small for a longer period of time, but that the source population has been substantial in size. This ROH pattern is probably indicative of small isolated or island populations, as was also found to a smaller degree in relatively small human populations [28].

The fact that cumulative ROH size was dominated by large ROHs in Asian pigs indicates that the population size has only recently reduced and these pigs are derived from large source populations (Figure 2). The European wild boars, by contrast, showed a more uniform distribution of ROHs relative to chromosomal position (Figure 7), consistent with a long lasting low effective population size. Findings by Scandura et al. [23] confirmed that genetic diversity in current European wild boars (apart from those on the Italian Peninsula) was mostly affected by glacial bottlenecks. Nothnagel et al. stated that recent population growth in humans over the last ∼200 years has not significantly contributed to genomic ROH distribution [13]. For the current study on pigs, ROHs did appear to be affected, both by population growth, as previously larger ROHs are broken down by recombination in a non-uniform distribution (Figure 6 and Figure 7), and population decline, as new ROHs are formed. These different results may be a consequence of the demographic histories of the two species, since humans have had a global population expansion, but wild boars and pigs experienced severe (local) population reductions. Thus it seems like ROHs reflect both the recent past and current status of a population as well as distant population history, and are very susceptible to population dynamics.

South-East Asia has been pinpointed as the center of origin of *Sus scrofa* ([21], [22] Frantz LAF et al., unpublished data). Thus, it is not surprising that the estimated haplotype diversity in the Asian source populations was found to be higher than in European populations. This phenomenon is also seen in the (out of Africa) human genetic patterns, where linear relationships exist between nucleotide diversity and distance to source of origin [29], [30]. Likewise, the westward migration across Eurasia by *Sus scrofa* likely involved numerous founder events. These events are expected to result in lower genetic diversity in Western Eurasian populations. Moreover, recent studies have found evidence for more intensive bottlenecks in Europe compared to Asia due to Pleistocene glaciations [21]. Thus, we expect a degradation of the overall genetic diversity in European populations compared to Asian populations. This phenomenon was most apparent in the nucleotide diversity outside ROHs (Figure 5). The demographic decline of most European wild boar populations did not seem to cause a decline in genetic variation within these populations according to [23]. Here we show, however, that patterns of ROH distribution as well as nucleotide diversity outside of ROHs are consistent with a long and ongoing history of small local effective population sizes.

In the breeding industry, a possible consequence of artificial selection is a reduced effective population size and associated genetic diversity. Pig breeders, however, are generally concerned about retaining sufficient genetic variability to maintain a good selection response in the future [31]. Based on microsatellite markers and quantitative measures of genetic diversity such as the expected heterozygosity (H), [32] suggested that the genetic variation within domesticated pig breeds is not significantly lower than within the wild boar genome. This is confirmed by our findings in that European wild boars contained more ROHs and lower nucleotide diversity outside ROHs compared to European domesticated pigs. Even though the Duroc and Hampshire breeds cluster within one clade with the European wild boars (Figure S1) their ROH pattern and nucleotide diversity are typical for the European breeds (Figure 5). The ROH pattern is a signature of similar treatment of the breeds. In closed populations, nucleotide diversity outside ROHs, named π-out, should reflect ancient haplotype variation that was present in the ancestral population and should not be strongly affected by (recent) occurrences of autozygosity. The difference in overall π and π-out was highest in European commercial pigs. This suggests high haplotype diversity in the ancestral population, despite the more recently formed populations with smaller effective population size that result in ROHs that reduce the overall nucleotide diversity π. This may be explained by a recent admixture between European and Asian breeds. Indeed, during the industrial revolution, Asian pigs were imported to Europe to improve local pigs. Such introgression of Asian pig genomes in Europe is expected to have increased overall genetic diversity in European pigs compared to their wild counterpart. The hybridization could have introduced distinct haplotypes resulting in fewer IBD tracts and probably higher variation. The higher nucleotide diversity may be a consequence of improvement of European breeds by hybridization with Asian pigs [33], [34], [35], showing the strong influence of outcrossing. Among the Dutch wild boars that were analyzed using the Illumina porcine 60K beadchip data, two showed a highly distinct ROH pattern compared to other individuals from the same population (Figure 4B). A previous study identified these two individuals as being recently introgressed with domesticated pigs [36]. These individual-specific ROH patterns and the relatively high nucleotide diversity in the European breeds underline the importance of parental ancestry for the levels and pattern of variation in the genome of the offspring.

We were able to use the same re-sequence based methodology to study the ROH pattern in a few closely related *Sus* species. The most outstanding individuals in terms of low nucleotide diversity and number of ROHs were found in this outgroup. The bearded pig *Sus barbatus*, most widespread in Borneo, had a minimal genomic ROH coverage. This indicates that the population has been large enough to avoid consanguineous matings for a substantial (recent) time period. Interestingly, the genome of *Sus cebifrons* displayed few ROHs, despite the fact that this species only occurs on a few small islands in the Phillipines. Nevertheless, the average ROH size was the largest of all individuals, and it showed a low π-out indicative of small ancient population sizes. As this species is confined to a few small islands, founder effects could explain the low observed π-out. The species *Sus verrocusus* had the lowest nucleotide diversity and ROHsize. This indicates a very small current and past effective population size, consistent with its endangered status on the IUCN red list. The inbreeding could have intensified due to this individual coming from a zoo. Other factors, such as different mating system may also influence ROHs distribution. For example, the mating pattern is expected to be different in artificially managed populations than in natural populations. In addition, closely related species or even separate wild populations can have different hierarchical systems that strongly influence the effective population size [7]. For instance, the Chinese domesticated pigs cluster closer to the European pigs in Figure 5 than the European wild boar, although the phylogenetic tree displays a different clustering (Figure S1). This indicates that the pattern of haplotype variation is similarly shaped in domestic populations, despite having a mostly independent domestication history.

### Effects of recombination rate

From population genetic theory, the effects of linkage disequilibrium are important to understand variation in genomes [37], particularly in the presence of selection. Large parts of genomes seem to be under selection, and all functional sites in a genome are potentially under purifying selection [38] or adaptive evolution, even in non- transcribed regions [39]. The effects of selection and demography are expected to have an interaction with the recombination landscape in the genome, thereby shaping genome wide variation in individuals and populations. This interaction has so far been poorly studied even in species for which considerable genomic resources exist, and has been neglected in studies on genetic conservation [10].

ROH distribution over the chromosomes was found to be non-random in other mammals, including humans [13], [16], [28], [40]. The proportion of ROHs in the genome was much higher in pig than in any other species studied so far, with individuals containing ROHs in over 75% of their genome. The U-shaped distribution of recombination rate was more profound in pig compared to other mammals. Despite a high degree of conserved synteny between human and pig [41], it is surprising that this pattern is so pronounced in pigs compared to humans. Correlations between ROHs and LD exist for other species, and were also very strong in pigs, with a higher recombination rate outward of the central chromosomal regions and in short chromosomal arms [42]. We showed that heterozygosity is higher towards these peripheral regions, a phenomenon that was previously observed in pigs [43]. In humans, chromosome size seems to be an important determinant of ROH occurrence [13]. In pigs the occurrence of ROHs was not proportionally higher on larger chromosomes, but seemed to be present throughout the genome and mostly influenced by the physical position on the chromosome, particularly in relation to local recombination rate. The higher abundance of small ROHs towards the telomeres probably stems from the central part of the chromosome being covered by the larger ROHs that have not been broken down due to lower recombination in this region. A bottleneck in the past with stable or on-going population growth ever since may lead to a more equal distribution of ROHs, as observed in the European wild boars.

Genomic features, GC content, nucleotide diversity and recombination rate, were all correlated and displayed similar U-shaped chromosomal distributions in the porcine genome (Figure 6A–6D). This has important implications for the probability of autozygosity in different chromosomal areas. Large ROHs appeared significantly more often in regions with low recombination. The difference in pattern of ROHs between European domesticated pigs and European wild boars is probably related to more continuous inbreeding in European wild populations, which have only expanded their population and range in the past 60 years [36]. Breed formation in European pigs has likely resulted from hybridization of different domestic and wild origins, including pigs originating from Asia. Pig populations, defined as breeds or commercial lines, are likely to have had an effective population size, in many cases measured in tens rather than hundreds or thousands, over the past decades. Many traditional breeds have been marginalized, with very small breeding stock [44]. Even the commercial pure lines, particularly the boar lines that are usually applied in three- or fourway crosses to generate the finisher pigs that go to slaughter, are often kept closed with small effective population size. Larger ROHs were therefore mostly found in regions of low recombination rate in domesticated pigs, because time since formation has been short. Small ROHs are thought to be present in a population longer than large ROHs and are more often shared among individuals than large ROHs. The rationale behind this is that recombination will degenerate large ROHs with time, but in regions with little or no recombination, small ROHs will be retained. Therefore, despite the time factor, these non-recombining regions will preserve ROHs when created, while recently originated large ROHs may occur randomly in the genome before they are degradated. The number of ROHs and the size distribution of the ROHs are therefore important determinants of recent and more historical population bottlenecks and inbreeding events.

### Effects of gene content and selection

Coding sequences are generally GC-rich regions in mammals, including pigs [45], [46]. We found a correlation between ROH occurrence and GC content in the genome, but not between global gene content and ROHs. The apparent lack of gene enrichment in ROHs suggests no direct correlation to the ROHs identified in our study and selection acting on genes. However, it is possible that some of the ROHs overlap with non-coding functional elements such as cis-regulatory modules. Although a few regions were identified where loss of genetic diversity may have been the result of selection, our study suggests that vast majority of the ROHs are likely to be neutral. The occurrence and distribution of ROHs, therefore, are mainly shaped by the interaction of past demographic events and recombination rate.

For the Large White breed, of which 13 individuals were sequenced, only 54 Mbp was found to be homozygous across all individuals combined, a fraction of the total of the genome embedded in ROHs across the same population. The total sum of homozygosity for each individual, therefore, is much larger than it is for the population. In the Large White breed, some genes were found in the homozygous regions that are possibly under positive selection associated with traits of commercial interest, such as fast reproduction. These genes are, however, found in regions that are large (many Mbp in size). In other populations, such as the European wild boar, the cumulative shared homozygous regions are much shorter and not always carrying genes, which could indicate that, despite the high degree of homozygosity in individual genomes in wild populations, selective sweeps may not be very common. Some overlapping ROHs may contain selected genes that are associated with defense mechanisms and adaptations to novel environments, but the fact that no genes were found in many overlapping ROH regions between the wild boars elucidates the stochasticity of ROH occurrence. We conclude that only a small fraction of the ROH-containing regions in pigs are homozygous due to positive selection.

### Conclusion

Our study shows that the formation of ROHs is mainly influenced by past demographic events and local recombination rate. This finding implies that inbreeding and recombination rate may act together in regions containing genes, mimicking selection. Genes in regions of low recombination, therefore, are at higher inbreeding risk, and could experience more rapid fixation. This phenomenon can have drastic influence on the fitness of individuals in small populations.

The genome-wide correlation of ROHs with the local GC content and recombination rate highlights the importance of genomic features such as recombination rate for autozygosity predictions. Many diploid species are likely to be heterogeneous in genome-wide recombination rate. This means that estimating inbreeding coefficients from effective population size, pedigrees, and even genetic data such as microsatellite genotype data [12], [47], does not completely measure the proportion and distribution of IBD homozygosity. Therefore of risk of inbreeding depression is underestimated. In addition, in a selective sweep analysis such local genomic regions of low recombination may wrongly be interpreted as being under selection.

Our re-sequencing based methodology to determine genomic variation implements genomic ROH distribution as a separate variable to nucleotide diversity. We show that the method is applicable even to closely related non-model species. Therefore, its utility exceeds species boundaries and combines different characteristics of diversity in diploid organisms. We show that both population demography and recombination landscape influences genomic ROH occurrence and these factors should both be taken into consideration when designing genetic conservation strategies in wild and domesticated species. We suggest more research on the genome-wide mechanisms that prevent the negative effects of inbreeding by influencing the localization of ROHs.

## Materials and Methods

### Experimental setup

A total of 52 animals were selected for re-sequencing and genotyped on the porcine 60K SNPchip. We re-sequenced one individual per species of *Sus barbatus*, *Sus celebensis*, *Sus cebifrons* and *Sus verrucosus*, and one warthog (*Phacochoerus africanus*) representing one of the closest relatives outside the genus *Sus*. Within *Sus scrofa*, the five European pig breeds Duroc, Hampshire, Large White, Pietrain, and Landrace were represented by 4, 2, 13, 5 and 5 individuals, respectively. A total of six animals from European wild boar populations from four distinct populations from the Netherlands, France and Italy were included as a separate group, as well as five Asian wild boars (two from North China, two from South China and one from a small population originated from a Japanese island). Finally, seven Chinese pigs, four from the Meishan breed, two from the Xiang breed and one from the Jianquhai breed were selected to represent the variation within Asian domesticated pigs. An additional 241 individuals from *Sus scrofa* populations, for which individuals were sequenced, were genotyped for SNP assay based ROH analysis. Because of ascertainment bias and paucity of segregating SNPs on the 60K chip for other Suids than *Sus scrofa*, no other Sus species were genotyped (Figure S5).

### DNA extraction, SNP genotyping, and library preparation

DNA was extracted from whole blood by using the QIAamp DNA blood spin kit (Qiagen Sciences). Every DNA sample was checked for quantity and quality on the Qubit 2.0 fluorometer (Invitrogen) and run on a 1% agarose gel. SNP genotyping was performed on the Illumina Porcine 60K iSelect Beadchip [48]. DNA from all individuals was diluted to 100 ng/ul and genotyped according the IlluminaHD iSelect protocol. Data was analyzed using Genome Studio software (Illumina Inc.). In case of re-sequencing, library construction and re-sequencing of the individual samples was performed with 1–3 ug of genomic DNA according to the Illumina library prepping protocols (Illunima Inc.). The library insert size was aimed for 300–500 bp and sequencing was performed with the 100 paired-end sequencing kit.

### Sequencing and SNP discovery

All selected individual pigs from domesticated breeds and wild populations were completely sequenced to ∼8× depth (details on coverage in Table S1). Reads were trimmed to a phred quality >20 and minimum length of both pairs of 40 bp, and the quality trimmed reads were aligned to the Sus scrofa reference genome build 10.2 [21] using the unique alignment option of Mosaik Aligner (V. 1.1.0017) to avoid erroneous called SNPs due to copy number variations and repeats. The data has been deposited to the Sequence Read Archive (SRA) at EBI, under accession number ERP001813 (link: http://www.ebi.ac.uk/ena/data/view/ERP001813). SNPs were called and filtered with mpileup from the SAMtools (V.0.1.7 r510) software package [49] with default settings for diploid organisms. Additional filtering was applied to the called variants with VCFtools (minDP = 7; minDP calling a SNP = 2; maxDP = ∼2*average coverage; INDELs excluded). By setting the minimum depth to call a SNP to 7× and only consider a base sufficiently covered at 7×, we reduce the number of missed variants. Nucleotide diversity was calculated for bins of 10 kbp over the entire genome within each individual. “SNPbin” is the SNP count per 10 kbin, corrected for the number of bases within that bin that was not covered enough for the VCFtools filtering, so that the eventual SNP count per bin (SNPbin) is proportional to 10.000 covered bases. SNPcount = total number of SNPs counted in a bin of 10 kbp. DP = coverage in bp/bin (per base at least depth of 7× and maximum of ∼2*average coverage). Binsize = 10.000. Correction factor = DP/binsize. SNPbin = SNPscount/Correction factor.

### Phylogenetic tree construction

A phylogenetic tree was constructed for the 52 re-sequenced individuals. We genotyped these individuals on the Illumina Porcine 60K iSelect Beadchip. Based on these genotypes, an IBS similarity matrix was created using Plink 1.07 [50]. Subsequently a neighbor joining hierarchical clustering was performed using the program Neighbor available from the Phylip package [51].

### ROH definition

Regions of homozygosity were extracted for all autosomes of the 52 re-sequenced individuals. Sex chromosomes were excluded as their recombination landscape is known to be different from the autosomes and the genetic map resolution for the X-chromosome differed from the autosomes in pig. Moreover, males and females should have been treated differently when the X-chromosome would have been included, and such analysis falls outside the scope of this paper. Autozygosity (a genomic region that was inherited from a common ancestor by both parents, and therefore indicates a certain level of relatedness) can typically be traced back in the genome as a ROH. The autozygous stretch is eventually broken into shorter pieces by recombination. A region of homozygosity is a genomic stretch that contains less variation in an individual than is expected based on the genomic average. Autosomal homozygous stretches (ROHs) for the re-sequenced individuals were determined using a sliding window approach. SNPs were counted in bins of 10 kbp, and those bins that fall into a window of 10 consecutive bins with a total SNP average below the genomic average were extracted in both the forward and reverse orientation. All neighbor bins were concatenated to form homozygous stretches. Out of this selection, only those stretches that contained a SNP count below a set threshold were considered part of a true homozygous stretch. The threshold was set to a SNP count of maximum 0.25 times the genomic average, with a maximum absolute value per stretch of the false discovery rate plus the mutation rate (μ = 2.5*10^−8^) because in some cases that exceeded the value of 0.25 times the genomic average. The false discovery rate was calculated based on the homozygous loci genotyped on the Illumina Porcine 60K iSelect Beadchip [48] that were called as a heterozygous locus in our database by vcftools (average ∼1.78 per bin). The rationale behind a threshold for heterozygosity rather than no heterozygote allowance is based on the thought that mutations in originally autozygous stretches may mask autozygosity over time. The genome-wide heterozygosity of an individual expresses the present variation in the population, and the associated chance that a certain autozygous stretches will reunite. The sequenced individuals varied greatly in the genomic heterozygosity and in population history. In addition, not all populations were sampled equally. Therefore the height of the threshold was based on the genomic average of the tested individual only, rather than the total set of individuals or an allele frequency-based likelihood of ROH occurrence. The threshold of 0.25 times the genomic average is based on permutations where the individual SNP distribution is randomized over all chromosomes. At a value of <0.25 times the genomic average, the observed ROH distribution deviated from the randomized distribution (see Figure S6). Local assembly or alignment errors were avoided as much as possible by relaxing the threshold for individual bins within a homozygous stretch, allowing for maximum twice the average SNP count in a bin, if the local maximum of 10 bins did not exceed 2/3 times the genomic average, and if the average of the ROH surrounding the presumed erroneous bin(s) still matched the previously mentioned criteria. Insufficiently covered bins (DP = <10%) were excluded from the SNP average calculations but were included in the ROH size determinations, with accepted ROHs containing maximum 2/3 uncovered bins and containing covered bins at both ends (example in Figure S7). In an analysis where the coverage of all individuals was lowered, we used a range of 5 thresholds for bin coverage (DP = <5, <10, <20, <50, <80%) and proportion of uncovered bins within a ROH (<1/4, <1/3, <1/2, <2/3, <3/4). We compared the outcomes with the highly covered individuals, and the errors in ROH size and abundance due to low coverage were minimized when thresholds of DP = <10% for bin coverage and <2/3 of missing ROH bins were used.

We genotyped 241 individuals on the Illumina Porcine 60K iSelect Beadchip for ROH detection (details in Table S2). ROHs were calculated with the Runs of Homozygosity tool in PLINK (v.1.07) with adjusted parameters (–homozyg-density 1000, –homozyg-window-het 1,–homozyg-kb 10, –homozyg-window-snp 20) [50]. Markers were filtered for call rate >95%. The homozygosity tool in PLINK v.1.07 does not include removal of MAF <0.05 or LD pruning when assessing ROHs. We aimed at keeping the ROH detection methods for the 60K data and genomic data as similar as possible in order to make sound comparisons. Therefore, no additional filtering for low allele frequencies was done, because sampling was unequal across populations and removing rare alleles could result in an overestimation of ROH in individuals from undersampled populations. No adjustments according to recombination rate were done because part of our goal was to analyze the influence of recombination rate on the occurrence of ROHs. For the re-sequenced animals, correlations with ROHs defined with PLINK were tested with the R (v.2.11.1) *cor* and *cor.test* functions.

### Population size estimations

Estimates of effective population size and split between the European (n = 2, from the Netherlands and Italy) and Asian (n = 2, from North and South China) wild boars were inferred using a HMM as implemented in PSMC [52] on copy number neutral fragments with a cumulative size of 1 Mbp, with a generation time of five years (g = 5) and default mutation rate/generation (μ = 2.5*10^−8^).

### Statistical analysis of the genome ROH distribution

All genomic features are based on the non-repeat masked *Sus scrofa* reference genome (build10.2). Values for GC content and nucleotide diversity were calculated for each relative chromosomal distance (0–1 with steps of 0.05) and averaged for all chromosomes. Based on the porcine genetic map [18] we estimated recombination rates based on the ratio of genetic and physical distances of neighboring markers within the relative bins, averaged over all markers in the bin. For comparisons with recombination rate in the human, mouse and cow genome we used the genetic distances and chromosomal sizes described by Myers, Shifman and Arias respectively [53], [54], [55]. Four groups (Asian wild, European wild, Asian breeds and European breeds) were analyzed separately and correlation coefficients for the relative ROHbin distribution within the groups and the genomic features were calculated and tested for significance by the R (v2.11.1) *cor* and *cor.test* functions with Pearson's product-moment correlation. The between-group differences in outside-ROH-nucleotide diversity, ROHnumber and ROHsize were tested with one-way analysis of variance in R(v2.11.1). Proportional differences of ROHs between groups and uniformity of ROHs over relative chromosomal position were tested with the χ2 test for proportions and goodness-of-fit in R. All plots were generated with the R (v.2.11.1) lattice package and Ubuntu OpenOffice 3.2.1.

### ROH and gene content

Each chromosome was divided into 20 equal sized segments and the relative gene content per segment was calculated. ROHs were grouped according to the three size classes and per class their relative distribution over these chromosomal segments were calculated. Correlations of gene content and ROH content were tested with corr.test in R.

All the annotated porcine genes from *Sus scrofa* (build 10.2 Ensembl release 67), were extracted using Biomart [56]. The distribution of genes over chromosomes was calculated in a similar way as the ROH occurrence. Each chromosome was divided in 20 equal sized stretches (thus each stretch representing 5% of the chromosome), the total number of genes per stretch was counted and expressed as relative gene content per stretch, proportional to the total gene content on the chromosome. Since the human genome is better annotated, all the human Ensembl orthologues of porcine genes were considered for the gene ontology analysis. BinGO v2.44 [57] a Cytoscape v2.8.3 [58] plugin was used to identify over-represented GO terms related to biological processes using the human annotation as background. A hypergeometric test was used to assess the significance of the enriched terms and the Benjamini and Hochberg correction was implemented for multiple comparisons.

## Supporting Information

Figure S1 Phylogenetic tree for all 52 sequenced individuals. Distances are based on the genotypes on the Illumina Porcine 60K iSelect Beadchip. Three main clusters can be observed: The other Sus species originated from the South-East Asian Islands, The wild and domesticated Asian *Sus scofa* and the European wild and domesticated *Sus scrofa*. Branch lengths may be affected because of the ascertainment bias introduced by the focus on variable sites in European pigs during SNP chip construction.
(TIF)

Figure S2 Distribution of nucleotide diversity over chromosome 1. The x-axis displays the physical position on the chromosome in bp and the y-axis shows the corrected number of SNPs that was called in bins of 10 kbp. Data is shown for a Dutch wild boar from the Veluwe, for a pig from the European Pietrain breed, for a wild boar from North China and for a wild boar from a Japanese island.
(TIF)

Figure S3 Estimation of demographic history and population size with the Pairwise Sequentially Markovian Coalescent (PSMC). The x-axis displays the years back in time, and the y-axis shows the estimated effective population size N. Data is shown for Two Asian wild boars from North (red) and South China (green) , and two European wild boars from the Netherlands (purple) and France (blue).
(TIF)

Figure S4 Correlation between genomic gene content and ROH frequency. 4A. Distribution of gene content over relative chromosomal position, plotted for all chromosomes separately. Metacentric chromosomes are displayed in blue and acrocentric chromosomes in red. Relative gene content plotted against ROH frequency for small (4B), medium (4C) and large size ROHs (4D). ROH distribution is given for four groups: European breeds (red), European wild boars (green), Asian breeds (purple) and Asian wild boars (including the Japanese, orange).
(TIF)

Figure S5 Comparison between the genomic data and 60K data on ROH number and cumulative size. The x-axis displays the number of ROHs that was counted for each individuals, and the y-axis shows the cumulative size of ROHs per individual. Data is shown for Genomic data (blue) and 60K data (red). All 52 sequenced animals are included in the analysis. Only for the 60K data the names of the individuals are included, showing that for the non *Sus scrofa* species (Warthog, *Sus verrucosus, Sus cebifrons, Sus celebensis and Sus barbatus*) and for the Japanese wild boar (WB20U02) the number and size of ROHs based on the 60K data are overestimated compared to the number and size of ROH based on the Genomic data. This is probably due to the ascertainment bias that is introduced to the 60K data because the chip is constructed based on polymorphisms that are found in European pig breeds.
(TIF)

Figure S6 Example of ROH detection test where SNP distribution was randomized. The x-axis shows the number of SNPs, averaged over all bins within a ROH, relative to the genome-wide average number of SNPs in a bin. The length of the ROH in terms of consecutive bins is displayed on the y-axis. ROH calculation was executed as explained in the methods section, except for the cutoff of 0.25 times the genomic average. The red dots display the true distribution of ROH length and SNP count within an individual. The blue dots show the distribution after permutation. As can be seen in the plot, the true distribution and the distribution based on a randomized SNP dstribution over the genome differ significantly below a relative SNP count of 0.25 times the genomic average per ROH. Values below the cutoff are shown in orange for the true distribution, and lightblue for the randomized distribution.
(TIF)

Figure S7 Example of ROH calculation. The x-axis represents the location on the chromosome and the y-axis shown the corrected number of SNPs that were counter per bin of 10 Kbp. The blue dotted line represents the chromosomal average and the purple line 2* the average. The mutation rate μ = 2.5*10^−8^ ( = 0.0025 SNP per bin of 10 kbp) and the false discovery rate is 0.0002 (2 SNPs per bin). The maximum SNP count in a ROHbin is in this case 0.25*10 = 2.5, because (2+0.0025)<2.5. The star indicates one bin within a ROH with SNP count 20. Because the local maximum does not exceed 2* the average ( = 20) and the maximum average of 10 surrounding bins ( = (9*2.5 +20)/10) = 4.3) does not exceed 2/3 times the average ( = 6.67) the bin is included in ROH1. Because the bins between ROH1 and ROH2 locally do exceed this maximum, they are not considered as being part of a ROH.
(TIF)

Table S1 Summary statistics of all sequenced individuals. The first two columns “Background” and “Groups” define the background of the individuals. The individuals that are included in the European breeds belong to the Duroc, Hampshire, Landrace, Large White and Pietrain breeds. The Chinese breeds are Jianquhai, Meishan and Xiang. European wild boars are The Dutch, French and Italian individuals and the Asian wild boars come from Japan and China. The *Sus barbatus, Sus cebifrons, Sus celebensis, Sus verrucosus* and Warthog (*Phacochoerus africanus*) are clustered in the group other species. The column “Individual” displays the codes for each individual pig. The average ROH size in bp within an individual is shown in column 4. The total number of ROHs detected within an individual is displayed in column 5. Column 6 shows the average nucleotide diversity in the genome of an individual, outside ROHs. The seventh column displays the average coverage in read depth for each sequenced individual. The last column shows the relative coverage of the genome of each individual, for which each base has at least a read depth of 7 and a maximum read depth of 2 times the average coverage.
(XLS)

Table S2 Summary statistics of all individuals genotyped on the Illumina Porcine 60K iSelect Beadchip. The first two columns “Background” and “Groups” define the background of the individuals. The total number of ROHs detected by PLINK is shown in the fourth column. The last column displays the total sum of ROHs in the genome.
(XLS)

Table S3 List of GO-IDs that were overrepresented in the Largewhite ROHs. Genes from all genomic regions that were homozygous in all Large Whites were extracted. The GO-IDs in column 1 were overrepresented in these ROH regions (p-values in column B, Benjamini and Hochberg corrected p-values in column C). The description of the corresponding biological process is shown in column D and all genes that contributed to the GO group are listed in column E.
(XLS)

Table S4 List of GO-IDs that were overrepresented in the European wild boar ROHs. Genes from all genomic regions that were homozygous in all European wild boars were extracted. The GO-IDs in column 1 were overrepresented in these ROH regions (p-values in column B, Benjamini and Hochberg corrected p-values in column C). The description of the corresponding biological process is shown in column D and all genes that contributed to the GO group are listed in column E.
(XLS)
