# Supplementary figures and images for: Regions of Homozygosity in the Porcine Genome: Consequence of Demography and the Recombination Landscape
Source: PLoS Genet. 2012 Nov 29;8(11):e1003100. doi: 10.1371/journal.pgen.1003100 (PMC3510040; doi:10.1371/journal.pgen.1003100)

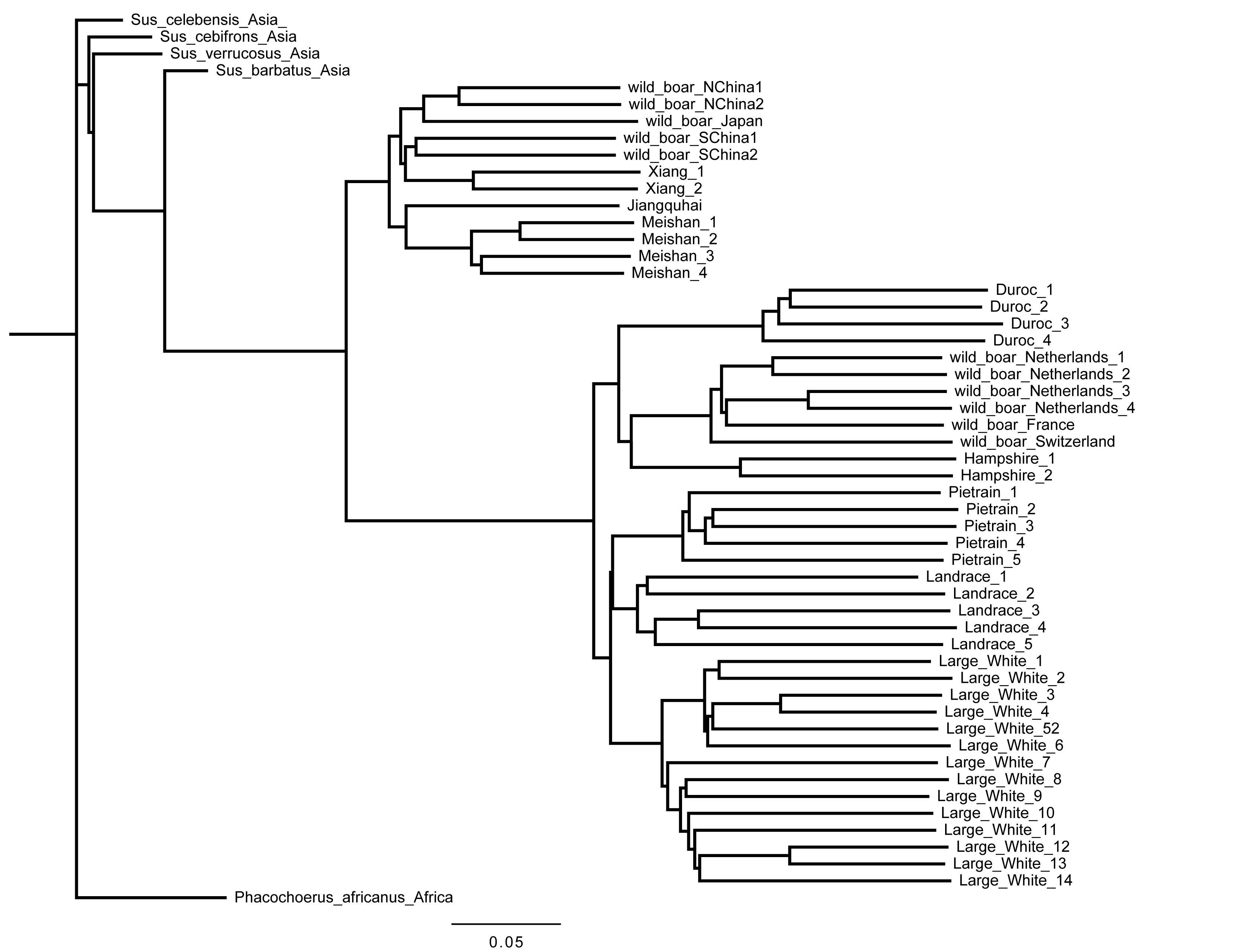

Supplement: Figure S1 — Phylogenetic tree for all 52 sequenced individuals. Distances are based on the genotypes on the Illumina Porcine 60K iSelect Beadchip. Three main clusters can be observed: The other Sus species originated from the South-East Asian Islands, The wild and domesticated Asian Sus scofa and the European wild and domesticated Sus scrofa. Branch lengths may be affected because of the ascertainment bias introduced by the focus on variable sites in European pigs during SNP chip construction. (TIF) [file pgen.1003100.s001.tif]

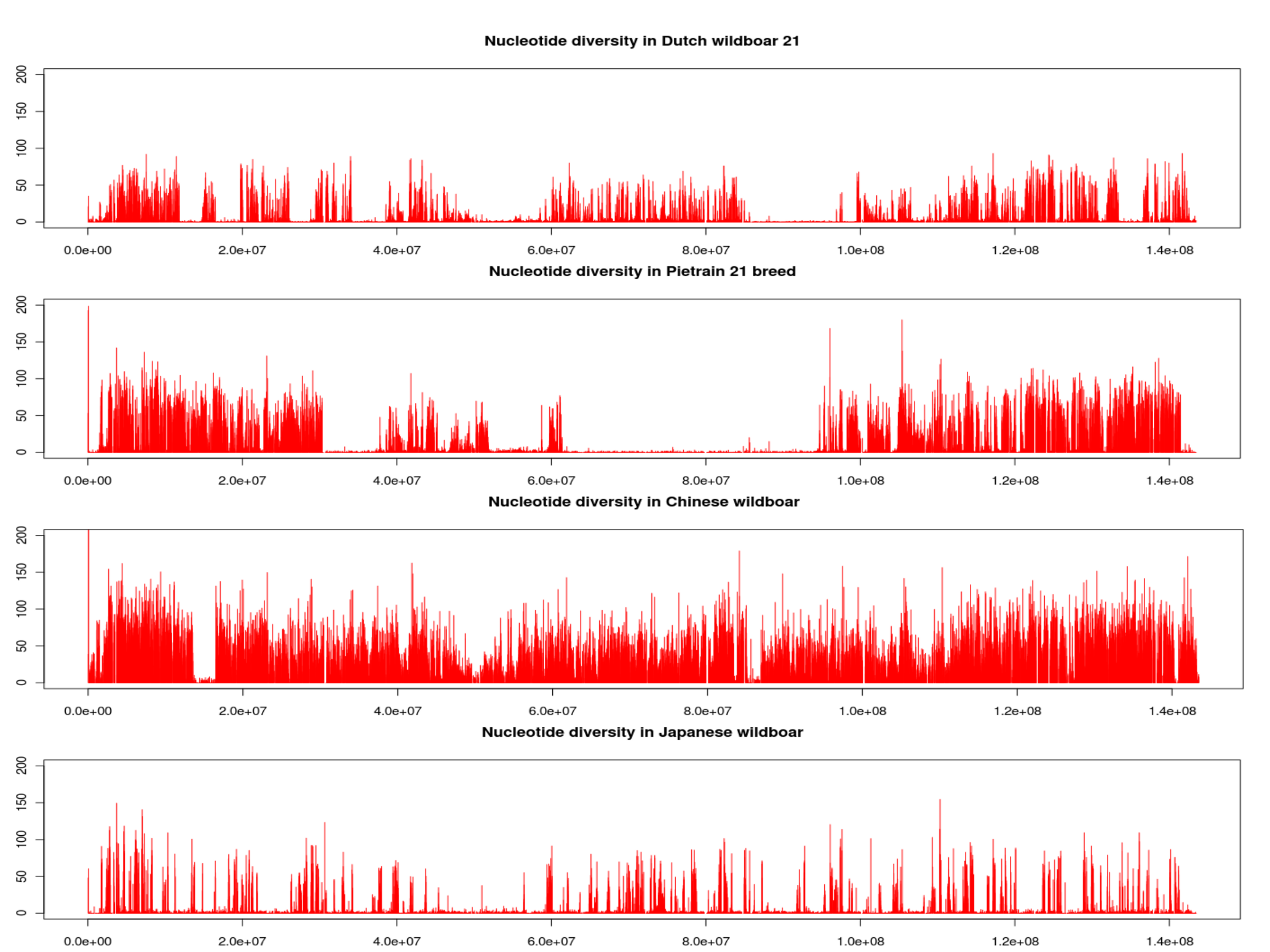

Supplement: Figure S2 — Distribution of nucleotide diversity over chromosome 1. The x-axis displays the physical position on the chromosome in bp and the y-axis shows the corrected number of SNPs that was called in bins of 10 kbp. Data is shown for a Dutch wild boar from the Veluwe, for a pig from the European Pietrain breed, for a wild boar from North China and for a wild boar from a Japanese island. (TIF) [file pgen.1003100.s002.tif]

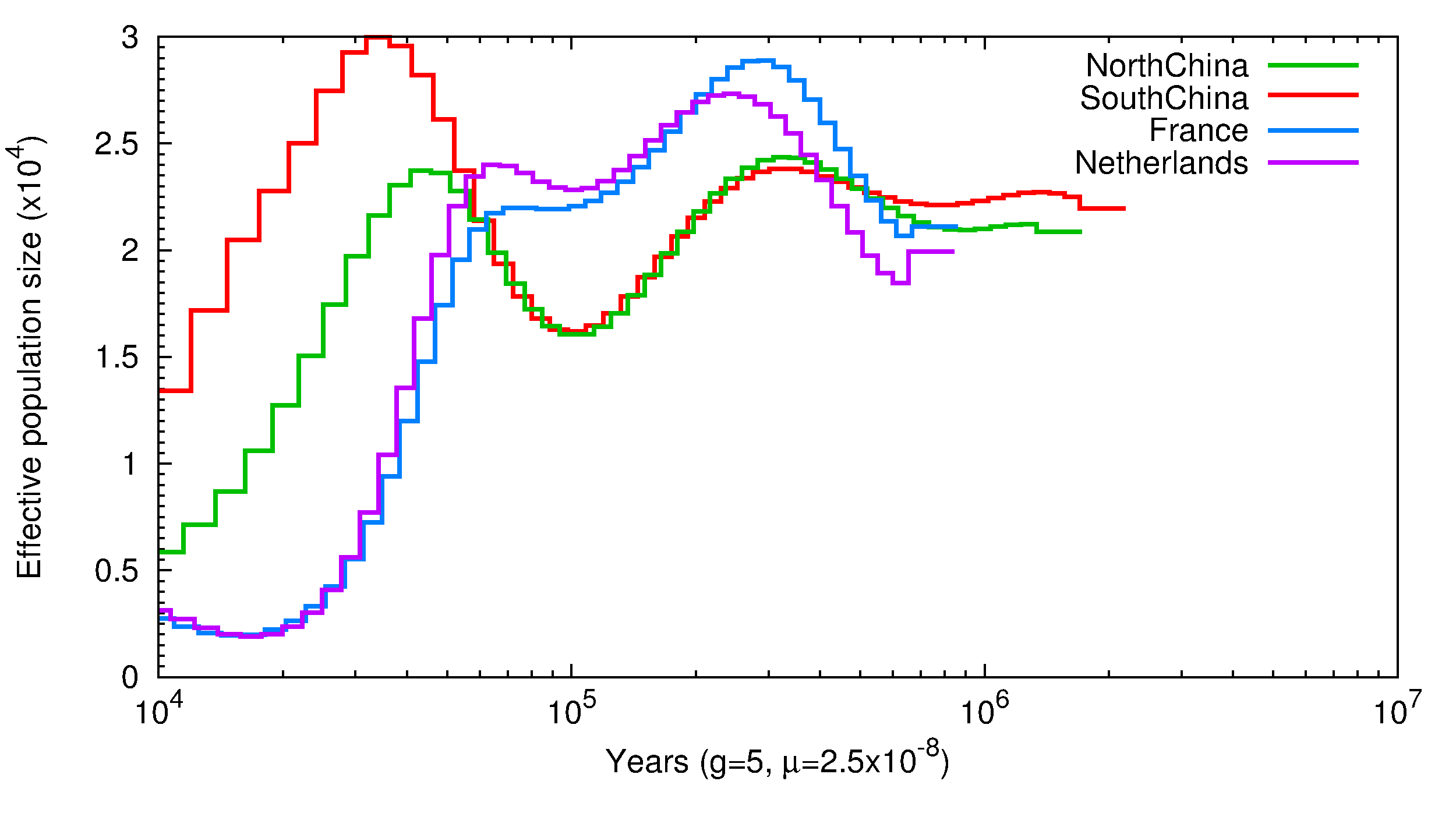

Supplement: Figure S3 — Estimation of demographic history and population size with the Pairwise Sequentially Markovian Coalescent (PSMC). The x-axis displays the years back in time, and the y-axis shows the estimated effective population size N. Data is shown for Two Asian wild boars from North (red) and South China (green) , and two European wild boars from the Netherlands (purple) and France (blue). (TIF) [file pgen.1003100.s003.tif]

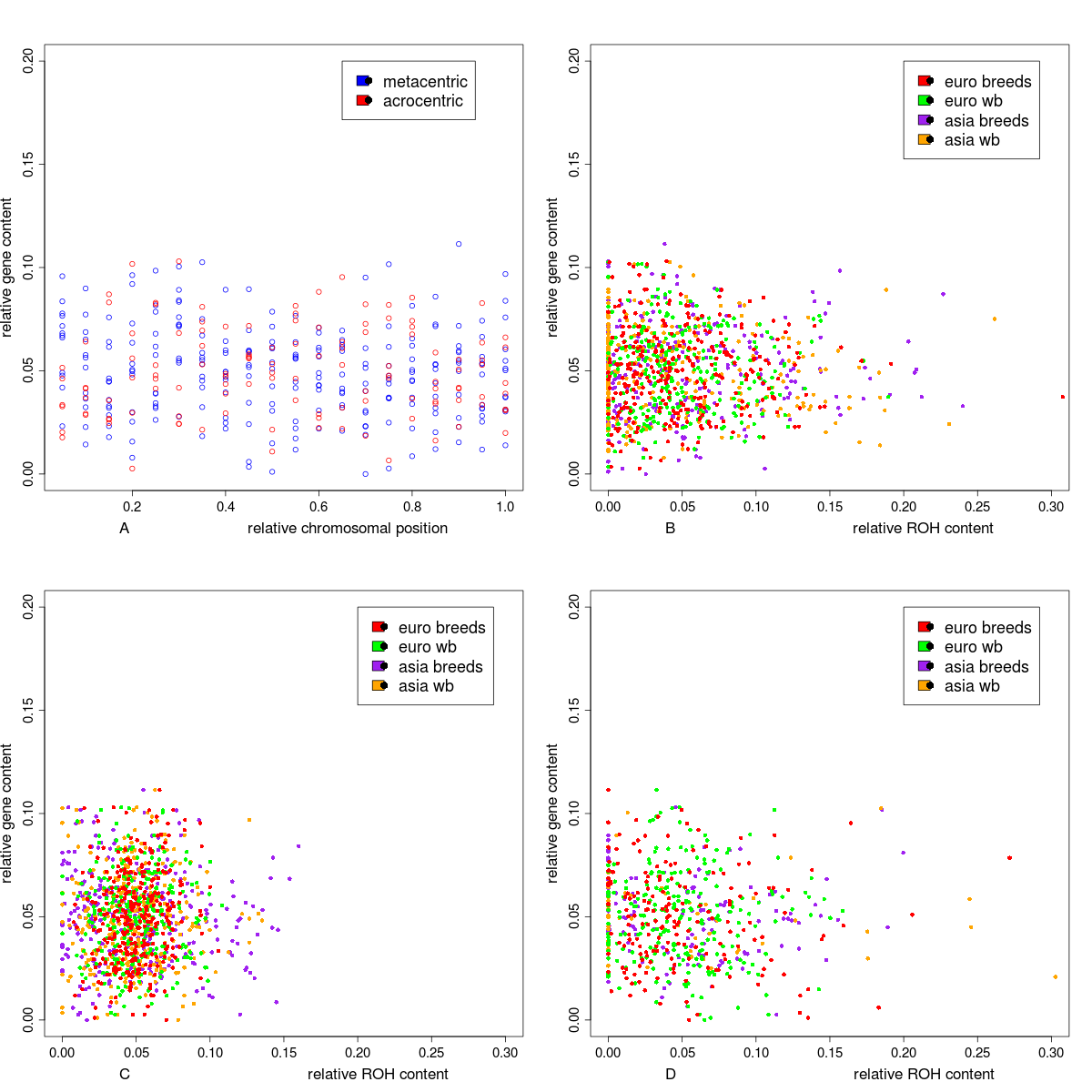

Supplement: Figure S4 — Correlation between genomic gene content and ROH frequency. 4A. Distribution of gene content over relative chromosomal position, plotted for all chromosomes separately. Metacentric chromosomes are displayed in blue and acrocentric chromosomes in red. Relative gene content plotted against ROH frequency for small (4B), medium (4C) and large size ROHs (4D). ROH distribution is given for four groups: European breeds (red), European wild boars (green), Asian breeds (purple) and Asian wild boars (including the Japanese, orange). (TIF) [file pgen.1003100.s004.tif]

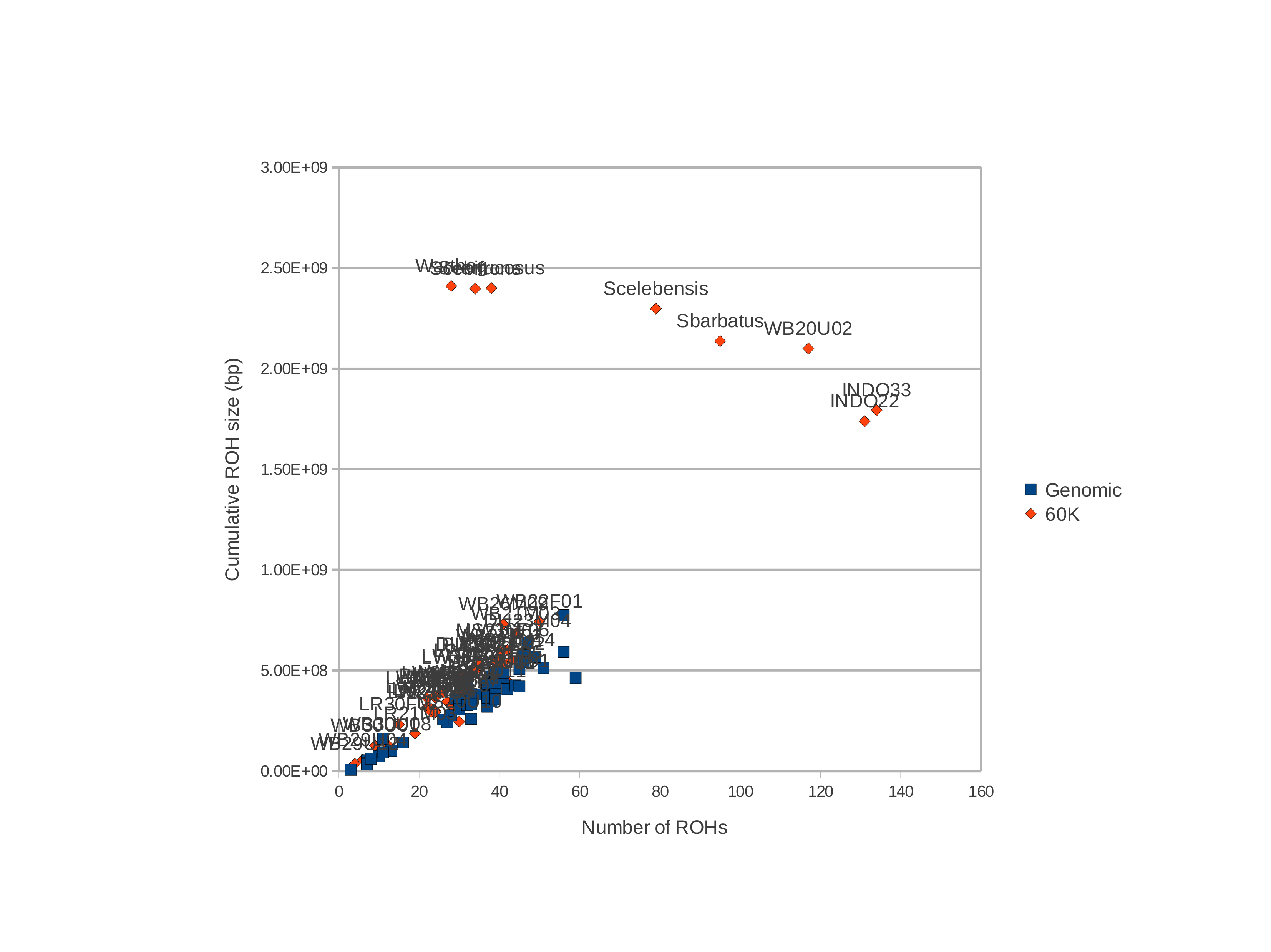

Supplement: Figure S5 — Comparison between the genomic data and 60K data on ROH number and cumulative size. The x-axis displays the number of ROHs that was counted for each individuals, and the y-axis shows the cumulative size of ROHs per individual. Data is shown for Genomic data (blue) and 60K data (red). All 52 sequenced animals are included in the analysis. Only for the 60K data the names of the individuals are included, showing that for the non Sus scrofa species (Warthog, Sus verrucosus, Sus cebifrons, Sus celebensis and Sus barbatus) and for the Japanese wild boar (WB20U02) the number and size of ROHs based on the 60K data are overestimated compared to the number and size of ROH based on the Genomic data. This is probably due to the ascertainment bias that is introduced to the 60K data because the chip is constructed based on polymorphisms that are found in European pig breeds. (TIF) [file pgen.1003100.s005.tif]

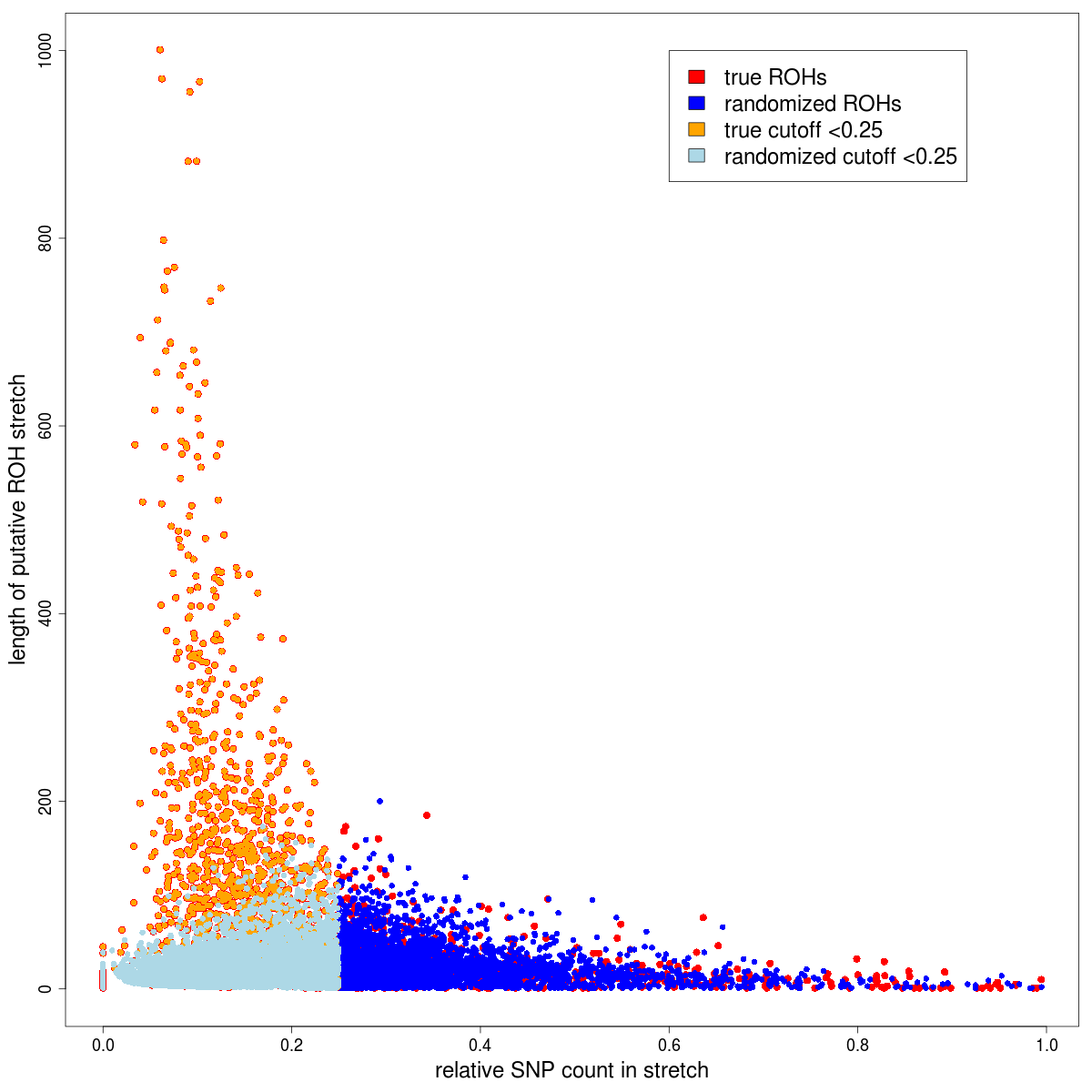

Supplement: Figure S6 — Example of ROH detection test where SNP distribution was randomized. The x-axis shows the number of SNPs, averaged over all bins within a ROH, relative to the genome-wide average number of SNPs in a bin. The length of the ROH in terms of consecutive bins is displayed on the y-axis. ROH calculation was executed as explained in the methods section, except for the cutoff of 0.25 times the genomic average. The red dots display the true distribution of ROH length and SNP count within an individual. The blue dots show the distribution after permutation. As can be seen in the plot, the true distribution and the distribution based on a randomized SNP dstribution over the genome differ significantly below a relative SNP count of 0.25 times the genomic average per ROH. Values below the cutoff are shown in orange for the true distribution, and lightblue for the randomized distribution. (TIF) [file pgen.1003100.s006.tif]

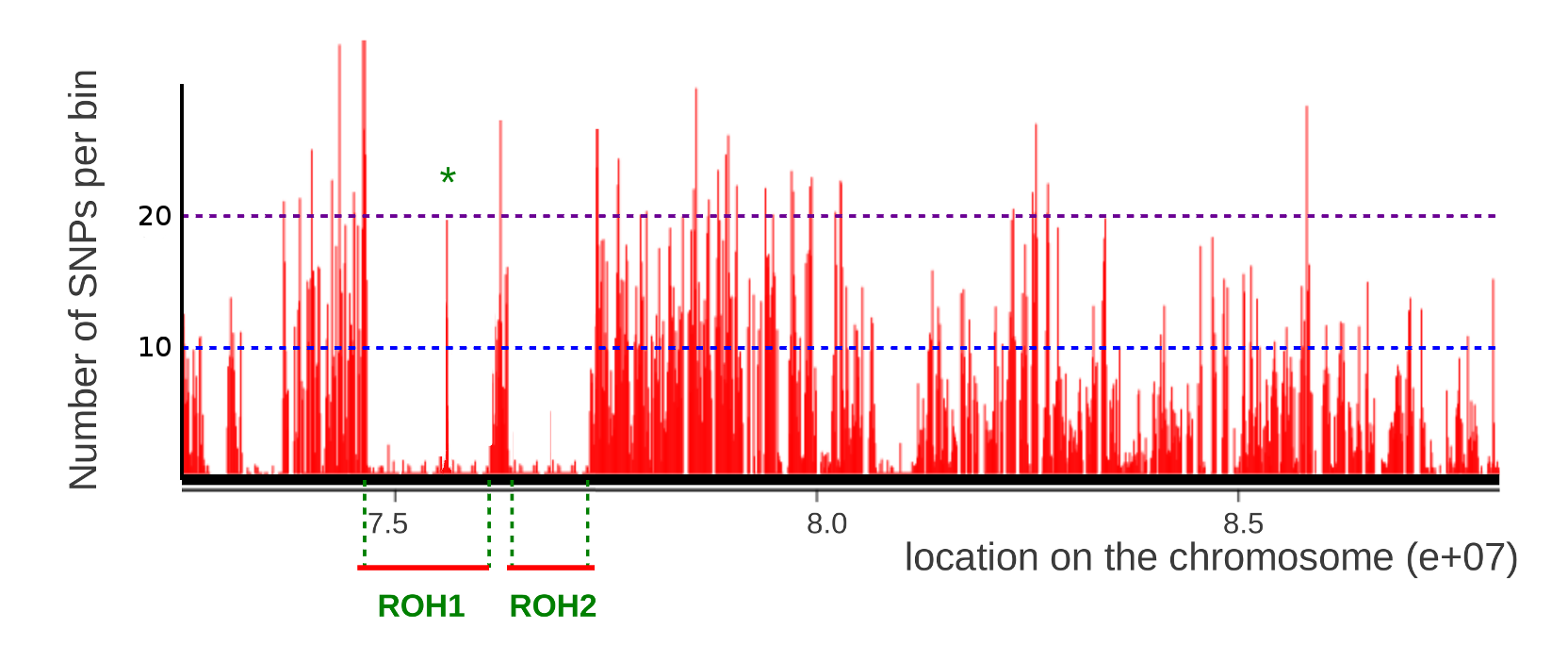

Supplement: Figure S7 — Example of ROH calculation. The x-axis represents the location on the chromosome and the y-axis shown the corrected number of SNPs that were counter per bin of 10 Kbp. The blue dotted line represents the chromosomal average and the purple line 2* the average. The mutation rate μ = 2.5*10−8 ( = 0.0025 SNP per bin of 10 kbp) and the false discovery rate is 0.0002 (2 SNPs per bin). The maximum SNP count in a ROHbin is in this case 0.25*10 = 2.5, because (2+0.0025)<2.5. The star indicates one bin within a ROH with SNP count 20. Because the local maximum does not exceed 2* the average ( = 20) and the maximum average of 10 surrounding bins ( = (9*2.5 +20)/10) = 4.3) does not exceed 2/3 times the average ( = 6.67) the bin is included in ROH1. Because the bins between ROH1 and ROH2 locally do exceed this maximum, they are not considered as being part of a ROH. (TIF) [file pgen.1003100.s007.tif]
